# Stoichiometry of Carbon, Nitrogen and Phosphorus in Shrub Organs Linked Closely With Mycorrhizal Strategy in Northern China

**DOI:** 10.3389/fpls.2021.687347

**Published:** 2021-09-07

**Authors:** Shuang Yang, Zhaoyong Shi, Menghan Zhang, Yang Li, Jiakai Gao, Xugang Wang, Dehong Liu

**Affiliations:** ^1^College of Agriculture, Henan University of Science and Technology, Luoyang, China; ^2^Luoyang Key Laboratory of Symbiotic Microorganism and Green Development, Luoyang, China; ^3^Henan Engineering Research Center of Human Settlements, Luoyang, China

**Keywords:** stoichiometry, mycorrhizal status, mycorrhizal types, shrub organs, Northern China

## Abstract

Mycorrhizal strategies include mycorrhizal statuses and mycorrhizal types, which are important reflections of the functional characteristics of ecosystems. The stoichiometry of carbon, nitrogen, and phosphorus in plant organs is an important part of ecosystem functions, which has an important impact on the nutrient cycle of the ecosystem. The concentration of carbon, nitrogen, and phosphorus played a crucial role in ecosystem functioning and dynamics. The purpose of this study is to provide theoretical basis and data support for improving the properties of global terrestrial ecosystems by exploring the impact of mycorrhizal strategies on the stoichiometry of C, N, and P in different shrub organs. In this study, stoichiometric patterns of carbon (C), nitrogen (N) and phosphorus (P) in different shrub organs under different mycorrhizal status or types were analyzed at 725 samples across Northern China. Results showed that in different mycorrhizal status, the highest carbon concentration in shrub organs appeared in the facultatively mycorrhizal (FM) mycorrhizal status, and the highest nitrogen concentration appeared in the Non-mycorrhizal (NM) mycorrhizal status. Under different mycorrhizal types, the nitrogen concentration in the shrub organs under the arbuscular mycorrhiza (AM) mycorrhizal type was the highest, and the phosphorus concentration under the ecto-mycorrhiza (ECM) mycorrhizal type was the highest. In the OM or FM mycorrhizal status, the concentrations of C, N, and P in the stems and leaves increase with the increase of the concentrations of C, N, and P in the roots. In the NM mycorrhizal status, the N concentration in the stems and leaves increases with the increase of the N concentration in the roots. Under AM, AM+ECM, and ECM mycorrhizal type, the concentrations of C, N, and P are closely related in roots, stems and leaves. The content of plant nutrients in different organs is closely related. It turned out that mycorrhizal statuses or types are able to alter the allocation of C, N, and P in different organs, and the relationships of C, N, and P among different organs are able to present different trend with the varying of mycorrhizal statuses or types.

## Introduction

The stoichiometry in plant leaves, stems, and roots is able to characterize the nutrient restriction status of nutrient elements (Wassen et al., 2005). Carbon (C), nitrogen (N) and phosphorus (P) are considered to be important stoichiometry because these elements are the basic elements that form the structure and function of all living things, which coupled strongly in their biochemical processes (Vrede et al., 2004). Carbon, which accounts for about 38% of plant dry matter, is an indispensable element in the growth and development of plants (Vrede et al., 2004). Leaf nitrogen concentration plays a vital role in photosynthesis, plant production, and litter decomposition (LeBauer and Treseder, 2008). Phosphorus is an important part of energy storage and cell structure, and it is critical for energy conversion, respiration, and photosynthesis (Sterner and Elser, 2002; Vrede et al., 2004). Moreover, stoichiometric characteristics of C, N, and P affect ecosystem functions and nutrient cycling (Lü et al., 2013). Stoichiometry provides a framework for understanding the role of ecosystems and the balance of elements. Previous studies have shown that evolutionary history, environmental stress and plant functional groups are possible factors affecting the stoichiometry of C, N, and P in different organs (Craine et al., 2005; Kerkhoff et al., 2006; Li et al., 2010; Liu et al., 2010). Moreover, some studies have suggested that C, N, and P stoichiometry of plants are affected by many factors, including climate and soil (Yang et al., 2016; Zhang et al., 2019), legumes and non-legumes (Yang et al., 2014), arbuscular mycorrhiza (Chen et al., 2010), productivity (Tang et al., 2018), nitrogen deposition (Jing et al., 2017), latitude (Fang et al., 2019), and environmental conditions (Gong et al., 2018). However, it is still unknown that the effect of the complete mycorrhizal strategy functional group on the stoichiometry of C, N, and P. This study considers that different mycorrhizal statuses or types will also affect the stoichiometry in different plant organs.

Mycorrhiza is a combination of certain fungi and plant roots. Mycorrhizal fungi are important members of the plant microbiota and can form a symbiotic relationship with the root of most plants on the earth (Averill et al., 2019). More than 92% of plant species have mycorrhiza (Sizonenko et al., 2020). Because mycorrhizal fungi are ubiquitous in terrestrial ecosystems, they are an important part of the soil biota and affect where and how plants grow (Johnson et al., 2010). There is evidence that plant-mycorrhizal symbiosis is essential for maintaining biogeochemical cycles and ecosystem functions (van der Heijden et al., 2015). Fungi can’t only protect plants from pathogens, but also regulate the relationship between plants and water (Veresoglou and Rillig, 2012). According to reports, plants have different growth responses to different fungi species (Treseder et al., 2018). Mycorrhizal strategies are divided into mycorrhizal status and mycorrhizal types. The status of mycorrhiza is closely related to the leaf economic spectrum (Shi et al., 2020). The mycorrhizal status reflects the consistency of the colonization of fungi within a species (Menzel et al., 2016). A recent hypothesis suggests that the mycorrhizal types are able to be used as a predictive framework for carbon and nutrient cycles within and throughout ecosystems (Wurzburger and Brookshire, 2017). Different mycorrhizal types of plants have different nutrient acquisition strategies (van der Heijden et al., 2015). In recent years, the status and type of mycorrhiza have been the focus of attention in the interaction between plants and environmental factors. The relationship between the mycorrhizal statuses or types of plant species and other plant traits is important to the understand plant’s ecology and distribution. There have been many reports on the importance of mycorrhiza to forest ecosystems (Kubisch et al., 2016; Wu et al., 2019; Tarquin et al., 2021) and grassland ecosystems (Kriszta and Matthias, 2015; Maarten et al., 2018). A vast number of hypotheses explain the observed relationships between mycorrhizal association and plant ecology are available. However, it is unknown that the importance of mycorrhizal strategy to the shrub ecosystem. Therefore, this research will focus on whether the status or types of mycorrhiza have an effect on the stoichiometry of shrub organs and how they affect it.

Shrubs cover over 1.23 million square kilometers (equal to12.5% of national territory area) in China (Geological Publishing House, 2007). As the main plant type in Northern China, shrubs are the top-level vegetation adapted to the arid climate in Northern China (Yang et al., 2016), which are able to conserve water, regulate runoff, and improve soil fertility (Zhang et al., 2013). The shrubs are generally smaller than trees and are relatively more uniform in size among species, which weakens the “dilution effect” of carbon, nitrogen and phosphorus allocated to the structural components of woody plants (Kerkhoff et al., 2006). Exploring the stoichiometry in different organs of shrubs is essential to improve the properties of global terrestrial ecosystems. There have been previous studies on shrub stoichiometry (Yang et al., 2014; He et al., 2016; Zhang Q. et al., 2018), but the importance of mycorrhizal strategy to shrub stoichiometry is still unclear. Therefore, this study explores the stoichiometric characteristics based on shrubs, which is conducive to a deeper assessment in a larger ecological background. In this study, we explore the effects of mycorrhizal statuses or types on the content of C, N, and P among leaves, stems and roots and their relationships based on shrubs in Northern China. The purpose of this study is to explore the effects of mycorrhizal types or statuses on the distribution and relationship of carbon, nitrogen, and phosphorus among different shrub organs. The research aims to enhance the understanding of the stoichiometry of mycorrhiza in shrub organs by analyzing plant traits related to mycorrhiza development. Therefore, our hypotheses are: (1) Mycorrhizal statuses or types will alter the allocation of C, N, and P in different organs, (2) the relationship of C, N, and P among different organs is closely related to the mycorrhizal statuses or types.

## Materials and Methods

### Study Site and Investigation

This study collected 725 samples in Northern China between July and September (mostly July and August). A species that is always found to form mycorrhizas is considered to be obligately mycorrhizal (OM), while a species that is found to form mycorrhizas in one habitat but not in another habitat is considered to be facultatively mycorrhizal (FM). And a species is considered to be Non-mycorrhizal (NM) if it always not found to form mycorrhizas (Smith and Read, 2008). We classified all the plants with typical arbuscular mycorrhiza (AM) structures as AM type and others unable to form AMs as non-AM type, according to the method employed by Koele et al. (2012) in studying ectomycorrhizal effect on global foliar traits and Shi et al. (2020) in exploring the relationship between the worldwide leaf economic spectrum traits and mycorrhizal traits. The typical arbuscular mycorrhizal structure including arbuscule or vesicle was considered when AM type was identified. Ecto-mycorrhiza (ECM) and non-ECM are classified in the same way. The species forming both AM and ECM are classified as AM+ECM type. The mycorrhizal types of some plant species were determined this study, while others were ascertained according to the published literatures based on the method employed by Averill et al. (2019), Wang and Qiu (2006) and Shi et al. (2020). The references included mainly Wang and Qiu (2006); Akhmetzhanova et al. (2012), Li et al. (2011), and Shi et al. (2008). Based on these references the plant species were assigned a mycorrhizal status (OM, FM, or NM), and type (AM, ECM, and AM+ECM) (Supplementary Data Sheet 1). In total, out of 725 plant samples in this study, we obtained mycorrhizal information for 582 samples (80.28%) and analyzed a total of 543 samples (74.90%). This was due to the species with a small sample size (39 samples) were omitted from the analysis in order to ensure the rigor of mycorrhizal categories and the accuracy of the results. Out of these 473 (87.11%) were OM, 33 (6.08%) FM and 37 (6.81%) NM; 316 (66.81%) formed AM, 64 (13.53%) AM+ECM, 93 (19.66%) ECM.

We selected a 20 m × 20 m plot with relatively uniform species composition and community structure as the sample area to represent the shrub community at the site. Three 5 m × 5 m quadrats were arranged in the center and any two diagonal corners of the sample area to collect soil samples. We identified all individuals to the species level and determined the mycorrhizal type according to the species. In total, we analyzed 543 individuals of 49 shrub species from 37 genera and 17 families from 155 sites. The main species include *Corylus heterophylla, Ostryopsis davidiana, Prunus sibirica, Spiraea pubescens, Tamarix chinensis, Vitex negundo*, etc. Please refer to the Supplementary Data Sheet 1 for detailed species information. We determined the level of shrubs based on the base stem, plant height, growth environment and growth years of the shrub. We generally divided it into three levels: large, medium, and small, and took three individuals at each level. We had at most 17 levels and at least one level. We collected all the adult leaves capable of normal photosynthesis, all the stems except the new stems of the current year and the whole roots of each individual outside and adjacent to the quadrate. The collected plant samples were dried and ground in the laboratory to the next analysis and determination.

The soil nutrient status of the sampling site was characterized by soil nitrogen and soil phosphorus. We analyzed the soil N content and soil P content of 49 species in 155 locations. Among them, the variation range of soil nitrogen concentration was 0.13–18.03 mg/g, the average value was 1.75 mg/g, and the variation range of soil phosphorus concentration was 0.08–2.01 mg/g, the average value was 0.55 mg/g. The specific soil nutrient values were recorded in the Supplementary Data Sheet 1. The soil samples were collected in three one-meter-deep pits on the diagonal of each 5 m × 5 m quadrat. And then the nine soil samples from each sample area were mixed well. The soil samples were air-dried and the roots were removed and then were ground through a 100-mesh sieve in order to facilitate the next measurement. Please refer to Yang et al. (2014) for more detailed information on data collections and locations of the sampling sites.

### Determination of Carbon, Nitrogen, and Phosphorus Concentrations

The carbon and nitrogen concentrations in different shrub organs and total nitrogen concentrations of soil (STN) were analyzed using an elemental analyzer (2400 II CHNS; Perkin-Elmer, Boston, MA, United States) under 950°C for combustion then reduced to 640°C. The phosphorus concentrations in plant samples and total phosphorus concentrations of soil (STP) were measured using the molybdate/ascorbic acid method after H_2_SO_4_-H_2_O_2_ digestion.

### Data Analysis

Because the element concentrations of leaves, stems, and roots at each sampling point were average at the species level, the data was statistically summarized, and the average value of three individual plants from each level was calculated to represent the element concentration at the same point. We used the average value of all data to represent the element concentration under this mycorrhizal status or type and make a significant comparison of different element concentrations under different mycorrhizal types and statuses. At the level of *P* < 0.05, the concentration of C, N, and P in shrub organs was analyzed by one-way analysis of variance (ANOVA), and Tukey multiple comparison method was used to test the significance of the difference. All analysis of significance was performed using SPSS 23.0. The element concentration and stoichiometric ratio in different shrub organs were converted to logarithm 10, and then linear regression analysis was performed. All the figures were generated using EXCEL 2016 (microsoft office) or SPSS 23.0. Under AM, AM+ECM, and ECM mycorrhizal types, the effects of site, species, and their interactions on nine categories (leafC, leafN, leafP, stemC, stemN, stemP, rootC, rootN, and rootP) were examined by linear mixed effect models. Each category was treated as a response variable. Site, species and their interaction were fixed effects. Mycorrhizal type was considered as random effects. The analysis of linear mixed effect models were conduct in R 4.0.5^1^.

The relationships between soil nutrient concentration and shrub organs P concentration were analyzed used a bivariate correlation analysis. Pearson correlation coefficient and bilateral significance test were selected for these statistical analyses in SPSS 23.0.

In order to show that the distributions of C, N, and P contents varied with the mycorrhizal category, density histogram was performed using SPSS 23.0.

## Results

### Carbon, Nitrogen and Phosphorus Concentration in Shrub Organs Under Different Mycorrhizal Status

**FIGURE 1:**
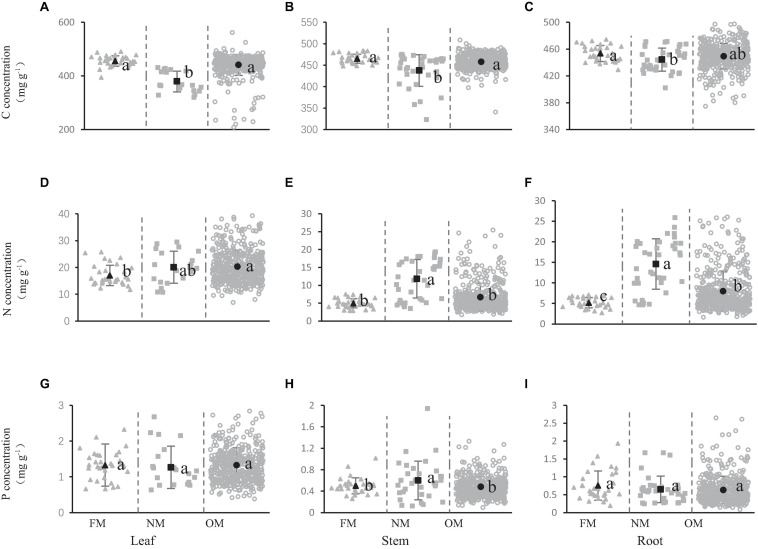
Carbon **(A–C)**, nitrogen **(D–F),** and phosphorus **(G–I)** concentration in shrub organs [leaf **(A,D,G)**, stem **(B,E,H)**, and root **(C,F,I)**] under different mycorrhizal status. The gray solid triangles represent the concentration of the elements in the FM mycorrhizal status, the gray solid squares represent the concentration of the elements in the NM, the gray open cycles represent the C, N, and P concentration of OM. The black dots represent the average value of C, N, and P concentration in this mycorrhizal status. Line bars show 95% confidence intervals. Letters above the line bars show the results of Tukey tests. Slopes with the same letters are not significantly different (*P* ≥ 0.05), while those with different letters are significantly different (*P* < 0.05).

The concentration of carbon, nitrogen, and phosphorus in shrub organs (leaves, stems, and roots) under different mycorrhizal status were presented in Figure 1. The carbon concentration in the leaves was 455.41, 379.02, and 440.73 mg/g in the different mycorrhizal status of FM, NM, and OM, respectively (Figure 1A). The carbon concentration in the stems was 464.88, 437.42, and 457.52 mg/g in the FM, NM, and OM mycorrhizal status, respectively (Figure 1B). The carbon concentration of leaves and stems in different mycorrhizal status of FM, NM, and OM presented the similar trend with the significant lower carbon concentration in the NM species than in OM and FM plants (Figures 1A,B). However, no remarkable difference was observed between FM and OM on carbon concentration either leaves or stems. In the roots, only a significantly higher carbon concentration was observed in FM shrubs than in NM shrubs. The carbon concentration in roots did not change markedly in FM and OM, and the same was true in NM and OM (Figure 1C).

Leaf nitrogen concentrations were 17.01, 20.07, and 20.35 mg/g (Figure 1D) in FM, NM, and OM species, respectively. And the nitrogen concentrations in the stem and root were 4.96, 11.85, and 6.68 mg/g (Figure 1E), 5.17, 14.60, and 8.01 mg/g (Figure 1F), respectively. The highest nitrogen concentration in the leaves was observed in OM species (OM plants have significantly higher leaf nitrogen concentrations than FM plants) (Figure 1D), however, this was not true for stems and roots, for which the highest nitrogen concentration was observed in NM species (Figures 1E,F). The nitrogen concentration in the stems of NM species was significantly higher than that of FM and OM species (Figure 1E). In the roots, the nitrogen concentration was highest in the NM, followed by the OM, and finally the FM, and the nitrogen concentration of roots was significantly different in the three mycorrhizal statuses (Figure 1F).

The phosphorus concentration in the stems was 0.50, 0.60, and 0.48 mg/g in FM, NM, and OM, respectively (Figure 1H). We found that NM species had markedly higher phosphorus concentration than FM and OM plants, while the FM did not exhibit significant variation of phosphorus concentration than OM (Figure 1H). In the leaves and roots of shrubs, there was no significant difference in the phosphorus concentration of the three mycorrhizal statuses (Figures 1G,I). The Figure 1 showed that different mycorrhizal status had an impact on the concentration of carbon, nitrogen, and phosphorus in the shrubs, but the impact was different.

### Carbon, Nitrogen and Phosphorus Concentration in Shrub Organs Under Different Mycorrhizal Types

**FIGURE 2:**
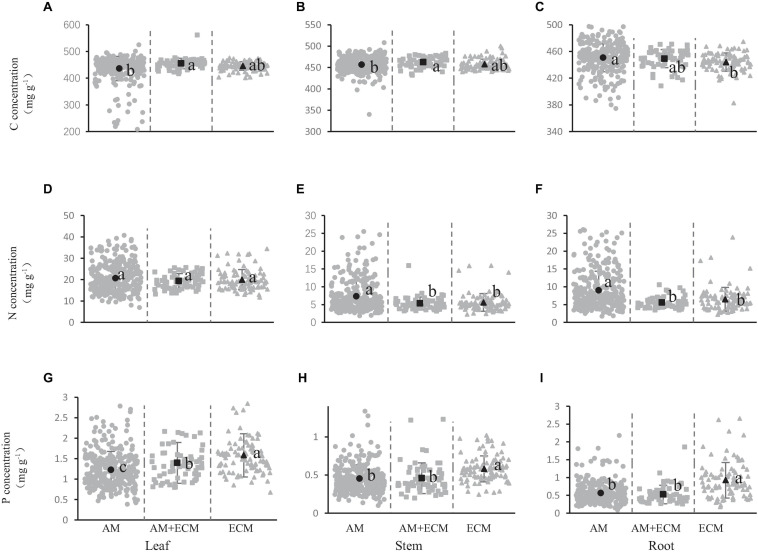
Carbon **(A–C)**, nitrogen **(D–F),** and phosphorus **(G–I)** concentration in shrub organs [leaf **(A,D,G),** stem **(B,E,H)**, and root **(C,F,I)**] under different mycorrhizal types. The gray solid cycles represent the C, N, and P concentration of AM species, the gray solid squares represent the element concentration of AM+ECM plants, and the gray solid triangles represent the element concentration of ECM mycorrhiza. The black dots represent the average value of C, N, and P concentration in this mycorrhizal types. Line bars show 95% confidence intervals. Letters above the line bars show the results of Tukey tests. Slopes with the same letters are not significantly different (*P* ≥ 0.05), while those with different letters are significantly different (*P* < 0.05).

Figure 2 showed the concentration of carbon, nitrogen, and phosphorus in shrub organs under different mycorrhizal types. Regarding mycorrhizal types, carbon concentration of leaves was significantly higher in AM+ECM plants (455.10 mg/g) than in AM species (436.14 mg/g) and the same pattern was observed in the stems, in which the carbon concentration in AM, AM+ECM and ECM was 456.50, 462.68, and 457.38 mg/g, respectively (Figures 2A,B). But in the leaves and stems, the carbon concentration did not exhibit significant variation among AM and ECM, AM+ECM, and ECM. In the roots, the carbon concentration was greater in AM compared to ECM, while both the carbon concentration in AM and ECM showed no significant difference from the carbon concentration in AM+ECM (Figure 2C).

There was no significant difference in nitrogen concentration between the three mycorrhizal types in shrub leaves (Figure 2D). The stem nitrogen concentration under AM, AM+ECM, and ECM was 7.28, 5.34, and 5.58 mg/g, respectively, and the nitrogen concentration in stems under AM was significantly higher than that under AM+ECM and ECM (Figure 2E). There was a similar rule in roots, for which the nitrogen concentration was 8.98, 5.55, and 6.46 mg/g, respectively (Figure 2F). However, we did not observed a significant difference of nitrogen concentration between AM+ECM and ECM in either stems or roots.

The leaf phosphorus concentration was 1.23, 1.39, and 1.58 mg/g (Figure 2G), and the stem phosphorus concentration was 0.45, 0.46, and 0.58 mg/g, respectively (Figure 2H), the root phosphorus concentration was 0.56, 0.53, and 0.93 mg/g, respectively (Figure 2I), in AM, AM+ECM, and ECM species. The leaf phosphorus concentration was significantly higher in ECM plants than in AM+ECM plants, and significantly higher in AM+ECM plants than in AM plants (Figure 2G). In the stems and roots of shrubs, there was a significant difference in the phosphorus concentration between ECM and AM, and there was also a significant difference in phosphorus concentration between ECM and AM+ECM (Figures 2H,I). The Figure 2 showed that the mycorrhizal types affected the concentration of carbon, nitrogen, and phosphorus in the shrub.

Linear mixed effect model analyses confirmed that, the concentration of carbon, nitrogen, and phosphorus in shrub organs varied with site and leafC, leafN, leafP, stemN, and rootN also varied with species. And the significant interactions were observed between the two variables (except stemC) (Table 1).

**TABLE 1:** The effect of site, species, and their interaction on the element concentration in shrub organs based on linear mixed effect models.

**Concentration**	**Site**		**Species**		**Site × Species**	
	***F***	***P***	***F***	***P***	***F***	***P***
leafC	7.19	<0.001	40.05	<0.001	10.96	<0.001
leafN	7.36	<0.001	58.94	<0.001	3.63	<0.001
leafP	6.93	<0.001	20.13	<0.001	2.30	<0.001
stemC	4.39	<0.001	0.93	0.33	1.21	0.20
stemN	4.78	<0.001	16.01	<0.001	2.45	<0.001
stemP	2.16	0.003	2.15	0.15	2.56	<0.001
rootC	5.08	<0.001	0.39	0.54	1.48	0.049
rootN	5.42	<0.001	37.02	<0.001	4.22	<0.001
rootP	2.10	0.007	0.76	0.38	1.76	0.008

*Site, species and their interaction are fixed effects. Mycorrhizal type is random effects. *P* < 0.01 means extremely significant, *P* < 0.05 means significant.*

### Effect of Ecto-Mycorrhizas and Arbuscular Mycorrhizas on Carbon, Nitrogen and Phosphorus Concentration in Shrub Organs

**FIGURE 3:**
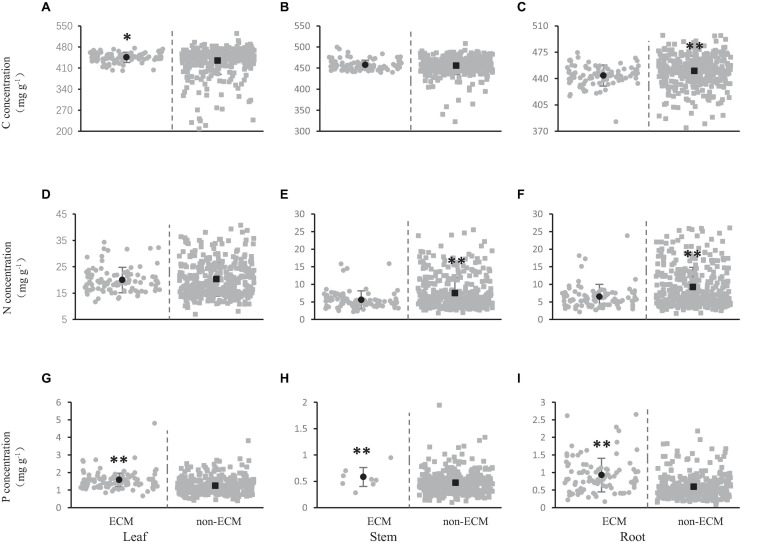
Effect of ecto-mycorrhizas on carbon **(A–C)**, nitrogen **(D–F),** and phosphorus **(G–I)** concentration in shrub organs [leaf **(A,D,G)**, stem **(B,E,H),** and root **(C,F,I)**]. The gray solid circles represent the C, N, and P concentrations of ECM species, and the gray solid squares represent the C, N, and P element concentrations of non-ECM species. The black dots represent the average value of C, N, and P concentration in this mycorrhizal types. The asterisks above the line bars show the results of Tukey tests at the level of *P* < 0.05. Two asterisks indicate extremely significant differences, and one asterisk indicates significant differences.

The carbon concentration in leaves, stems, and roots was 445.65, 457.38, and 444.03 mg/g, respectively, in shrubs where ecto-mycorrhizas were dominant; however, the carbon concentration in leaves, stems, and roots was severally 434.70, 455.45, and 450.25 mg/g, in shrubs with no ecto-mycorrhiza dominant (Figures 3A–C). The carbon concentration in the stems and leaves of shrubs with ecto-mycorrhiza was higher than that without ecto-mycorrhiza (Figures 3A,B), further the leaf carbon concentration with ecto-mycorrhiza was significantly higher than that without ecto-mycorrhiza (Figure 3A). The shrub roots showed the opposite trend, which was, the carbon concentration without ecto-mycorrhiza was significantly higher than that with ecto-mycorrhiza (Figure 3C).

The nitrogen concentration of leaves, stems and roots was 20.29, 7.50, and 9.21 mg/g without ecto-mycorrhiza, while the nitrogen concentration of leaves, stems and roots was 20.00, 5.58, and 6.46 mg/g when there was ecto-mycorrhiza (Figures 3D–F). In stems and roots, the nitrogen concentration without ecto-mycorrhizas was significantly higher than that with ecto-mycorrhiza (Figures 3E,F). But this significant difference did not appear in the leaves (Figure 3D).

Phosphorus concentration in leaves, stems and roots was 1.25, 0.47, and 0.59 mg/g without ecto-mycorrhiza. In contrast, the phosphorus concentrations of leaves, stems and roots was 1.58, 0.58, and 0.93 mg/g when there were ecto-mycorrhizas (Figures 3G–I). In the all shrub organs (leaves, stems and roots), the phosphorus concentration in when there was ecto-mycorrhiza was very significantly higher than when there was no ecto-mycorrhiza (Figures 3G–I).

**FIGURE 4:**
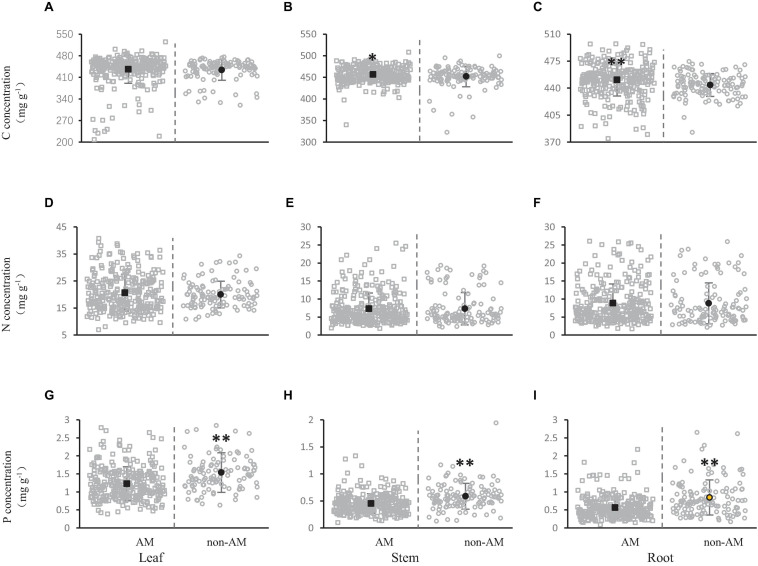
Effect of arbuscular mycorrhizas on carbon **(A,B)**, nitrogen **(D–F)** and phosphorus **(G–I)** concentration in shrub organs [leaf **(A,D,G)**, stem **(B,E,H),** and root **(C,F,I)**]. All gray open squares represent the concentration of C, N, and P in the shrub organs of AM plants, and all gray open circles represent the concentration of elements in non-AM plants. The black dots represent the average value of C, N, and P concentration in this mycorrhizal types. The asterisks above the line bars show the results of Tukey tests at the level of *P* < 0.05. Two asterisks indicate extremely significant differences, and one asterisk indicates significant differences.

Except for ecto-mycorrhizas, arbuscular mycorrhizas also affected the concentration of carbon, nitrogen and phosphorus in different shrub organs. The carbon concentration in leaves, stems and roots under non-AM or AM was 433.86, 451.92, and 444.07 mg/g (non-AM), 436.14, 456.50, and 450.65 mg/g (AM), respectively (Figures 4A–C). Carbon concentration of all organs (leaves, stems, and roots) was higher in AM than in non-AM. Further, we found that AM had markedly higher carbon concentration than non-AM in stems (Figure 3B), and the carbon concentration exhibited vary significant variation among AM and non-AM in roots (Figure 3C).

The nitrogen concentration in leaves, stems and roots was 20.00, 7.31, and 8.80 mg/g in non-AM, respectively. On the contrary, when AM is present, the nitrogen concentration of leaves, stems and roots was 20.67, 7.28, and 8.98 mg/g, respectively (Figures 3D–F). The leaves and roots of shrubs showed a tendency of higher nitrogen concentration when there was arbuscular mycorrhiza than when there was no arbuscular mycorrhiza. On the contrary, the stems of shrubs showed a tendency to have a higher nitrogen concentration when there was no arbuscular mycorrhiza than when there was arbuscular mycorrhiza. However, no significant difference was observed in nitrogen concentration in shrub organs (leaf, stem and root) (Figures 3D–F).

Phosphorus concentrations in leaves, stems and roots were 1.53, 0.59, and 0.85 mg/g without arbuscular mycorrhiza, and 1.23, 0.45, 0.56 mg/g when there was arbuscular mycorrhiza, respectively (Figure 3G–I). The leaves, stems and roots of shrubs all showed a tendency of higher phosphorus concentration when there was no arbuscular mycorrhiza than when there was arbuscular mycorrhiza. For the phosphorus concentration, extremely significant difference was observed among the AM and non-AM of all shrub organs (leaves, stems, and roots) (Figures 3G–I).

**FIGURE 5:**
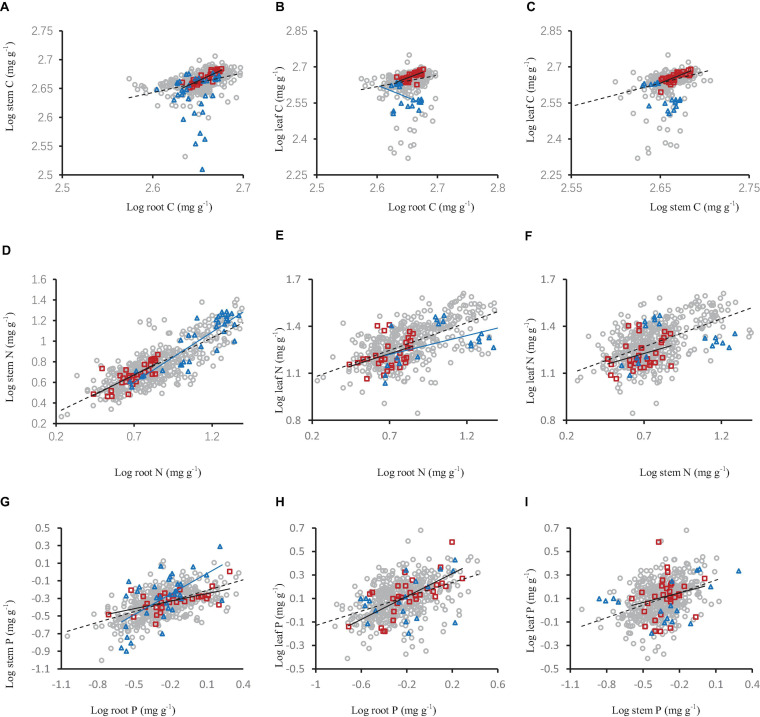
The relationships of carbon **(A–C)**, nitrogen **(D–F)** and phosphorus concentration **(G–I)** in shrub organs (leaf, stem, and root) under different mycorrhizal status. The gray open circle and broken line means OM, red square and black solid line means FM, blue triangle and blue line means NM.

**FIGURE 6:**
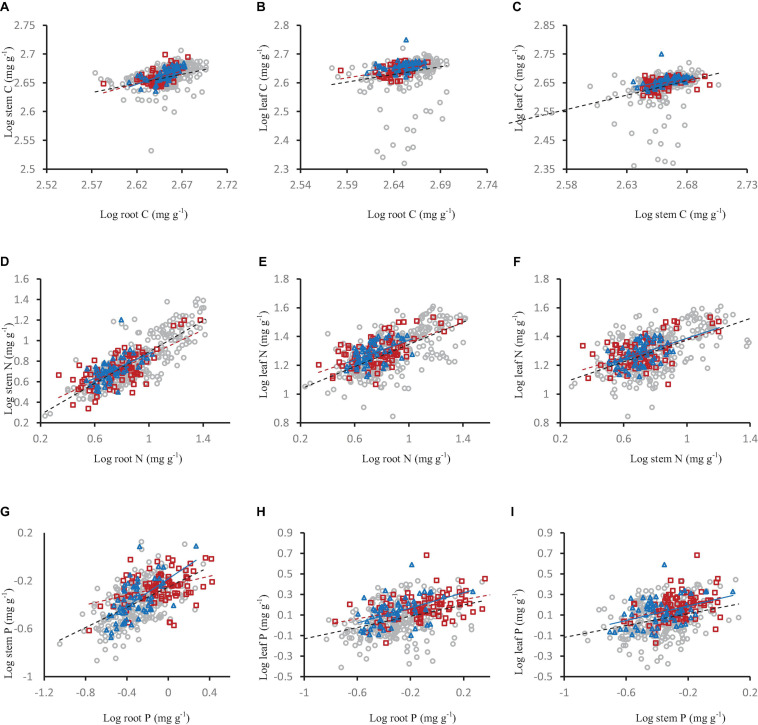
The relationships of carbon **(A–C)**, nitrogen **(D–F)** and phosphorus concentration **(G–I)** in shrub organs (leaf, stem, and root) under different mycorrhizal types. The gray open circle and black broken line means AM, the red square and red broken line means ECM, and the blue triangle and blue line means AM+ECM.

Figures 1–4 collectively showed that there were higher C, N, and P concentrations in leaves than in stems and roots. Mycorrhiza can promote the accumulation of carbon in shrub organs, and AM has a better promoting effect than ECM (Figures 1–4). On the contrary, mycorrhiza was not conducive to the absorption of nitrogen by shrub organs. Even so, plants dominated by AM can absorb more nitrogen than ECM-dominated plants. In addition, the nitrogen concentration of leaves, stems and roots under the AM+ECM treatment was the lowest, which indicated that the co-existence of AM and ECM may have a certain interaction and weaken the absorption of nitrogen by plants (Figures 1–4). ECM was significantly better than AM in promoting phosphorus uptake by shrubs (Figures 2–4). The contents of C, N, and P in different organs of shrubs were closely related. Generally speaking, plants with high nutrient contents in roots and stems also had higher nutrient concentrations in leaves (Figures 5, 6).

The C, N concentration in shrub organs varied greatly for both AM and non-AM plants, but the ranges were much larger for AM plants (except stem C) (Supplementary Figures 2–4). For example, leafC ranged from 208.89 to 525.34 mg/g for AM plants, while from 320.17 to 476.46 mg/g for non-AM plants; leafN ranged from 7.02 to 40.80 mg/g and from 10.84 to 34.35 mg/g for AM and non-AM plants, respectively; stemN from 1.86 to 25.53 mg/g and from 2.19 to 19.40 mg/g, respectively. And the C, N concentration in shrub organs varied greatly for both ECM and non-ECM plants, but the ranges were much larger for non-ECM plants (Supplementary Figures 5–7). For example, stemC ranged from 323.24 to 508.60 mg/g for non-ECM plants, while from 439.84 to 500.10 mg/g for ECM plants; stemN ranged from 1.86 to 25.53 mg/g and from 2.19 to 15.92 mg/g for non-ECM and ECM plants, respectively.

### The Relationships of Carbon, Nitrogen and Phosphorus Concentration in Shrub Organs Under Different Mycorrhizal Status and Types

Carbon, nitrogen and phosphorus concentration in different shrub organs under different mycorrhizal status or types had different relationships. In the OM and FM species, the stem carbon concentration and leaf carbon concentration increased significantly with the increase of root carbon concentration (Figures 5A,B), leaf carbon concentration increased significantly with the increase of stem carbon concentration (Figure 5C). This same pattern were also seen in nitrogen and phosphorus (Figures 5D–I). In the NM mycorrhizal status, the stem nitrogen concentration and leaf nitrogen concentration increased significantly with the increase of root nitrogen concentration, stem phosphorus concentration increased with the increase of root phosphorus concentration (Figures 5D,E,G).

Among different mycorrhizal types (AM, AM+ECM, and ECM), stem carbon concentration and leaf carbon concentration increased significantly with the increase of root carbon concentration, and leaf carbon concentration increased significantly with the increase of stem carbon concentration. There was the same pattern in nitrogen and phosphorus (Figure 6).

## Discussion

The significance of this study is to compare the concentration allocation and relationship of C, N, and P in shrub organs under different mycorrhizal statuses or types, in order to provide theoretical basis and data support for seeking the most suitable mycorrhizal status or types of shrubs to accumulate basic nutrient elements.

The analysis results of this study showed that the average carbon concentration in the organs of shrubs in northern China was similar to the reported value of global terrestrial plant carbon concentration (46.4 ± 3.21%, Elser et al., 2000). It is also similar to the carbon concentration of shrubs in southern China (Zhang et al., 2019). It showed that there was little difference in carbon concentration in shrubs between northern and southern China. The average concentration of nitrogen and phosphorus in stems and roots was lower than the reported value of global terrestrial plants (18.3 and 1.42 mg g^–1^, respectively; Reich and Oleksyn, 2004). This indicated that the concentration of nitrogen and phosphorus was relatively low in the stems and roots of Northern Chinese shrubs. Leaves have higher C, N, and P concentrations than stems and roots. Zhang’s results also showed that the concentration of nitrogen and phosphorus in photosynthetic organs (leaves) was higher than in non-photosynthetic organs (stems and roots) (Zhang Q. et al., 2018). This means that the nutrient concentrations in leaves of shrubs are higher than non-leaf organs. There were the same patterns in forests and grasslands (Zhao et al., 2016; Yu et al., 2017). This difference in nutrient distribution among plant organs may be related to leaf function. Leaves were the main organs for photosynthesis, respiration and water use, so they usually require higher nutrient concentrations than non-leaf organs to perform these physiological functions (He et al., 2016). For non-leaf organs such as stems and roots, their function was mainly to absorb and transport nutrient elements rather than metabolize, so they required lower nutrient concentration than leaves (Kerkhoff et al., 2006).

Mycorrhizal fungi are vital members of the plant microbiome and enhance the plant’s access to nutrients (Averill et al., 2019). Fungi provide nutrition and protection to plants, and the benefits from mycorrhiza depend on the type of mycorrhiza (Bennett et al., 2017). The mycorrhizal status of plants is one of the most typical underground features, because most terrestrial plants can form mycorrhizal symbiosis (van der Heijden et al., 2015). Whether plants are OM or FM, and which mycorrhizal type they form—AM, ECM or AM+ECM, it is of great significance to the species distribution of plants on the continental scale and their response to environmental gradients (Bueno et al., 2017). In addition, the large-scale model of plant mycorrhizal status can highlight the conditions that determine the importance of mycorrhizal symbiosis in the entire ecosystem (Menzel et al., 2016). ECM and AM are two different types of mycorrhizal fungi, which, respectively, represent the adaptation of plants to low soil nutrient availability and high soil nutrient availability (Averill et al., 2018). ECM plants were dominant in high latitude ecosystems with slow nutrient cycling, while AM plants were dominant in low latitude ecosystems with rapid nutrient cycling (Zhang H. Y. et al., 2018). In temperate zones, ECM plants are more conservative in their use of nitrogen and phosphorus than AM plants (Averill et al., 2019).

The concentration of carbon, nitrogen, and phosphorus played a crucial role in ecosystem functioning and dynamics (Kerkhoff et al., 2006; Vitousek et al., 2010). Therefore, it is necessary to study the concentration of C, N, and P. The carbon concentration in leaves, stems, and roots was highest under FM and lowest under NM. Moreover, the carbon concentration of FM plants in leaves, stems and roots was significantly the highest. The wider ecological niche and habitat of FM plants helped them cope with unstable and disturbed environments (Gerz et al., 2018). Except for roots, the carbon concentration under FM and OM was significantly higher than that under NM. It showed that the presence of mycorrhiza promote the accumulation of carbon by shrubs. Moreover, the highest carbon concentration in leaves and stems was under AM+ECM, and the highest carbon concentration in roots was under AM. The results showed that AM was better than ECM in promoting the carbon absorption of shrubs. AM plants commonly show better performance due to enhanced uptake of mineral nutrients and improved photosynthetic pigments (Smith and Smith, 2011). AM fungi were beneficial to enhance the shoot biomass and thus contribute to the accumulation of carbon (Chen et al., 2010). The symbiotic associations between AM fungi and plants can promote nutrient absorption by host plants (Smith and Read, 2008). Mycorrhizal fungal hyphae can obtain mineral nutrients by exploring soil volume and provide these nutrients to plants in return for carbon, so the formation of AM affects the distribution of carbon in plants (Chen et al., 2007; Smith and Read, 2008). Arbuscular mycorrhizal fungi can form a symbiotic associations with approximately 71% of vascular plants in all terrestrial ecosystems (Brundrett, 2017), the data in this article also accounts for more AM mycorrhizas (66.81%). AM-dominated vegetation has higher total plant diversity than ECM-dominated vegetation or NM-dominated vegetation (Aurele et al., 2020). AM can’t only help plants absorb nutrients (Bachelot et al., 2016), but can also improve plants’ drought, cold and salinity resistance (Wang et al., 2018; Li et al., 2019). Although roots did not affect the absorption of carbon, they affected the transport of carbon. The nitrogen concentration in stems and roots of shrubs was highest under NM, and the nitrogen concentration under NM in stems and roots was significantly higher than that under FM and OM. It showed that mycorrhiza was not good for shrubs to absorb nitrogen, this was due to the increase in plant biomass which dilutes the N (Chen et al., 2010). In stems and roots, when there was mycorrhiza, compared with the other two typical mycorrhizal types (AM+ECM, ECM), the nitrogen concentration under AM was the highest. It showed that although mycorrhizas were not conducive to the absorption of nitrogen by shrubs, plants with AM can absorb more nitrogen than ECM. The result of ECM had lower nitrogen concentrations than AM was in contrast with the result of most previous studies (Zhang H. Y. et al., 2018; Aurele et al., 2020), but it was in line with the result of Averill (Averill et al., 2019). Averill’s study showed that nitrogen in both green and senescent leaves varied with mycorrhizal types, and the nitrogen concentration in both of them showed ECM was less than AM (Averill et al., 2019). Plants related to AM mycorrhiza depend on the inorganic nitrogen resources of the soil, while ECM mycorrhiza will degrade and absorb the inorganic nitrogen in the soil (Averill et al., 2019). Previous studies have shown that the response of AM to nitrogen may be related to the availability of soil phosphorus (Johnson et al., 2003). The nitrogen concentrations in leaves, stems and roots were the lowest under AM+ECM, indicating that the co-existence of AM and ECM may produce some interaction to weaken the effect of nitrogen absorption by plants. For instance, as Aurele pointed out, the two main mycorrhizal types (AM and ECM) exhibited different, and often reciprocal, correlation (Aurele et al., 2020). The effect of ECM in promoting the absorption of phosphorus by shrubs was significantly better than that of AM. Many studies had shown that ECM exhibited greater phosphorus absorption relative to AM (Zhang H. Y. et al., 2018; Aurele et al., 2020). The reason why ECM was better than AM under certain conditions was that ECM changes the stoichiometry of the substrate by inhibiting soil nutrient mineralization, making them less available to AM (César et al., 2018). And the ECM generally exhibits positive plant soil feedback, compared with mostly negative feedback in the AM (Bennett et al., 2017), therefore, ECM may promote plant growth and thus facilitate the accumulation of phosphorus in plants. Theoretically, the symbiotic evolution of ECM may lead to the evolution of fungal phosphorus absorption characteristics, and the characteristics of phosphorus acquisition in ECM may be improved after this symbiotic evolution (Kohler et al., 2015). These all validate our first hypothesis that the statuses or types of mycorrhiza affects the allocation of carbon, nitrogen, and phosphorus in different plant organs, and this is consistent with research results of Kerkhoff et al. (2006), Yang et al. (2014), and Zhang Q. et al. (2018). This may be because different mycorrhizal types or statuses have different mechanisms to affect plants. It may also because different plant organs have different absorption mechanisms for nutrient elements, therefore, the allocation of carbon, nitrogen, and phosphorus in plant organs was different under different mycorrhizal statuses or types.

Non-AM plants had a larger range of phosphorus concentrations in leaves, stems, and roots than AM plants. We have observed that the phosphorus concentration in shrub organs (leaf, stem, and root) was extremely significantly higher in non-AM plants than in AM plants. We did further analysis to verify whether this result was related to the higher nutrient concentration in non-AM soil. The result was as expected, the soil nitrogen and phosphorus concentration of non-AM growth was extremely significantly higher than that of AM (Supplementary Figure 1). And the Pearson correlation analysis (Supplementary Table 1) showed that the phosphorus concentration in leaves, stems, and roots was significantly related to the soil phosphorus concentration. However, we did not find a correlation between stem P, root P and soil N. Research by Yang et al. (2016) also found a positive correlation between leaf P and soil P concentrations. Yang et al. (2016) speculated that this may be due to the fact that in the phosphorus-limited ecosystem [the concentration of leaf phosphorus in Northern China is significantly lower than in other parts of the world (Han et al., 2005)], plants may absorb phosphorus and precipitate it in the form of inorganic phosphorus when phosphorus in soil is abundant (Sterner and Elser, 2002), resulting a positive correlation between phosphorus in plant organs and phosphorus in soil. The mycorrhizal type was determined according to the species in this study. Mycorrhizal types affect the concentrations of carbon, nitrogen, and phosphorus (Zhang H. Y. et al., 2018; Averill et al., 2019), therefore, the concentration of carbon, nitrogen, and phosphorus will also vary with species. Changes in species composition may exacerbate the biogeographic patterns of nitrogen and phosphorus, leading to the species composition hypothesis (Peter and Jacek, 2004). Chagnon showed in his studies that different AM fungal species differ in their carbon requirements from host plants, phosphorus transfer to roots, carbon storage, and relative investment in both extracorrhizal and intracorrhizal biomass (Chagnon et al., 2013). Wang et al. (2015) showed that the concentrations of C, N, and P in stems and roots varied with species. The study by Zhang Q. et al. (2018) showed that there were significant differences in nitrogen and phosphorus concentrations between deciduous and evergreen, legume and non-legume plant species. Yang et al. (2014) also showed that there were significant differences between legumes and non-legumes. Many studies have shown that the concentration of carbon, nitrogen, and phosphorus varies with latitude (Peter and Jacek, 2004; Fang et al., 2019), these are consistent with our findings that the concentrations of carbon, nitrogen, and phosphorus in plant organs vary with site. This result may be caused by the different soil nutrient status, shrub types and water limitation status in different sites. More importantly, the effects of site and species interactions on carbon, nitrogen, and phosphorus concentrations in plant organs should be further studied.

The carbon, nitrogen, and phosphorus in different organs are interrelated, which has been testified by numerous studies (Zhang Q. et al., 2018). Zhang showed in his research that the content of plant nutrients in different organs was closely related (Zhang Q. et al., 2018). Essentially, leaf nitrogen and phosphorus are able to regulate the rates of carbon acquisition and use (Peter and Jacek, 2004). The allocation of nutrients among organs may be an important part of plant life history strategies. For example, plants with high nutrient content in roots and stems also have higher nutrient concentrations in their leaves. The reason for this phenomenon is that the underground parts of the plant (root) absorbs nutrients and transfer them to the aboveground parts (leaf, stem). The relationship among the different nutrients in plant can predict the changes of nutrient storage. An increase in the concentration and storage of nutrients in one organ will lead to a predictable increase in the concentration and storage of nutrients in other organs (Kerkhoff et al., 2006). In FM and OM species, the correlation between nutrients (C, N, and P) in stems and roots was stronger than that in leaves. This was closely related to the fact that stems and roots were non-photosynthetic organs with similar structures and functions (Fortunel et al., 2012). Furthermore, this suggested that mycorrhiza promoted the correlation between C, N, and P in the shrub organs. What we have observed that the increase in stem nutrients was accompanied by a similar increase in root nutrients agreed with Yang (Yang et al., 2014) and Zhang (Zhang Q. et al., 2018). Regardless of the mycorrhizal statuses or types, the nutrient concentration in the lower organ of the plant usually increases with the nutrient concentration in the adjacent upper organ (for example, root vs. stem, stem vs. leaf). This result was consistent with Brouwer’s hypothesis that plant organs compete for nutrients during growth, and the organs closest to the source of nutrients will be the first to obtain nutrients (Brouwer, 1983; Yang and Midmore, 2005). The nutrients will continue to be transported to the distant organs only after the organs closest to the nutrients have used up the nutrients (Yang and Midmore, 2005). Moreover, the mycorrhiza can significantly enhance the correlation between C, N, and P in different organs of shrubs.

In the future, it is still necessary to strengthen research on the effects of mycorrhizal status and types on different plant types, especially research on plant nutrient absorption, which plays an extremely important role in promoting better plant production and development. For future research, it will be quite interesting to compare the results of the study with current views on the mycorrhizal status.

## Conclusion

The mycorrhiza promoted the accumulation of carbon in shrub organs, but inhibited the absorption of nitrogen. AM was better than ECM in promoting the absorption of carbon by shrub organs. When there were mycorrhizas in plants, compared with ECM mycorrhiza and AM+ECM mycorrhiza, AM mycorrhiza had the most obvious effect in promoting the absorption of nitrogen by shrub organs, while ECM mycorrhiza had the most obvious effect in promoting the absorption of phosphorus by shrub organs. The statuses or types of mycorrhiza were able to change the relationship of carbon, nitrogen, and phosphorus in different organs of plants. In OM and FM mycorrhizal statuses and different mycorrhizal types, nutrient concentration between shrub organs were correlated. The distribution of nutrients between different organs was not independent.

## Data Availability Statement

The original contributions presented in the study are included in the article/Supplementary Material, further inquiries can be directed to the corresponding author/s.

## Author Contributions

SY and ZS: conceptualization, validation, writing—review and editing, and visualization. SY: methodology and writing—original draft preparation. ZS: software, investigation, data curation, supervision, project administration, and funding acquisition. SY, ZS, MZ, and YL: resources. All authors contributed to the article and approved the submitted version.

## Conflict of Interest

The authors declare that the research was conducted in the absence of any commercial or financial relationships that could be construed as a potential conflict of interest.

## Publisher’s Note

All claims expressed in this article are solely those of the authors and do not necessarily represent those of their affiliated organizations, or those of the publisher, the editors and the reviewers. Any product that may be evaluated in this article, or claim that may be made by its manufacturer, is not guaranteed or endorsed by the publisher.

## Footnotes

1
http://R-project.org/
