# Supplementary material for: Stoichiometry of Carbon, Nitrogen and Phosphorus in Shrub Organs Linked Closely With Mycorrhizal Strategy in Northern China
Source: Front Plant Sci. 2021 Sep 7;12:687347. doi: 10.3389/fpls.2021.687347 (PMC8453024; doi:10.3389/fpls.2021.687347)
Supplement: Supplementary file 2 [file Data_Sheet_2.docx]

**Supplementary Table S1** The bivariate relationships between the soil nutrient concentration and shrub organs P concentration

|  |  | soilN | soilP | leafP | stemP | rootP |
| --- | --- | --- | --- | --- | --- | --- |
| soilN | Pearson correlation | 1 | 0.471^**^ | 0.419^**^ | 0.082 | 0.088 |
|  | Significance (bilateral) |  | <0.0001 | <0.0001 | 0.370 | 0.341 |
|  | N(numbers) | 125 | 125 | 107 | 122 | 120 |
| soilP | Pearson correlation | 0.471^**^ | 1 | 0.466^**^ | 0.202^*^ | 0.408^**^ |
|  | Significance (bilateral) | <0.0001 |  | <0.0001 | 0.026 | <0.0001 |
|  | N(numbers) | 125 | 125 | 107 | 122 | 120 |
| leafP | Pearson correlation | 0.419^**^ | 0.466^**^ | 1 | 0.347^**^ | 0.421^**^ |
|  | Significance (bilateral) | <0.0001 | <0.0001 |  | <0.0001 | <0.0001 |
|  | N(numbers) | 107 | 107 | 112 | 109 | 107 |
| stemP | Pearson correlation | 0.082 | 0.202^*^ | 0.347^**^ | 1 | 0.342^**^ |
|  | Significance (bilateral) | 0.370 | 0.026 | <0.0001 |  | <0.0001 |
|  | N(numbers) | 122 | 122 | 109 | 127 | 123 |
| rootP | Pearson correlation | 0.088 | 0.408^**^ | 0.421^**^ | 0.342^**^ | 1 |
|  | Significance (bilateral) | 0.341 | <0.0001 | <0.0001 | <0.0001 |  |
|  | N(numbers) | 120 | 120 | 107 | 123 | 125 |

** indicates significant correlation at P<0.01 (bilateral); * indicates significant correlation at P<0.05 (bilateral).

**Supplementary Figure S1** Soil nitrogen and phosphorus concentrations of AM and non-AM plants

AM non-AM AM non-AM

The gray cycles represent the nutrient content in the soil in the AM mycorrhizal type, the gray squares represent the nutrient content in the soil in the non-AM mycorrhizal type**.** The black dots represent the average value of soil N, P concentration in this mycorrhizal types. The asterisk above the line bars show the results of Tukey tests. Two asterisks indicate extremely significant differences.


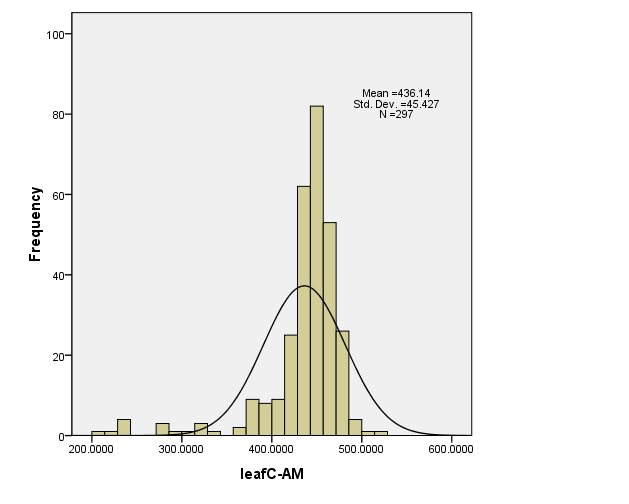

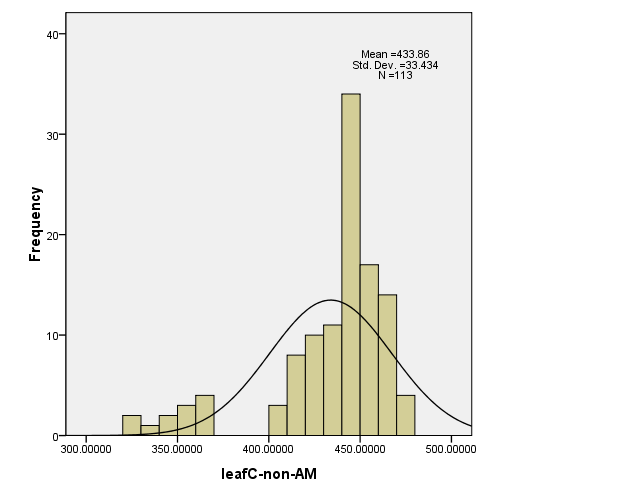


B

A


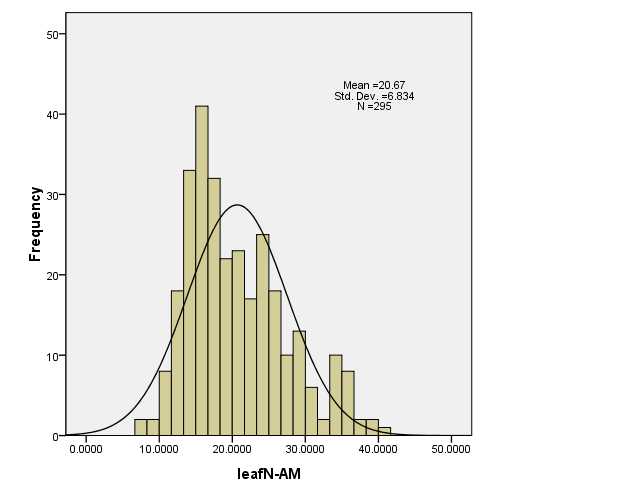

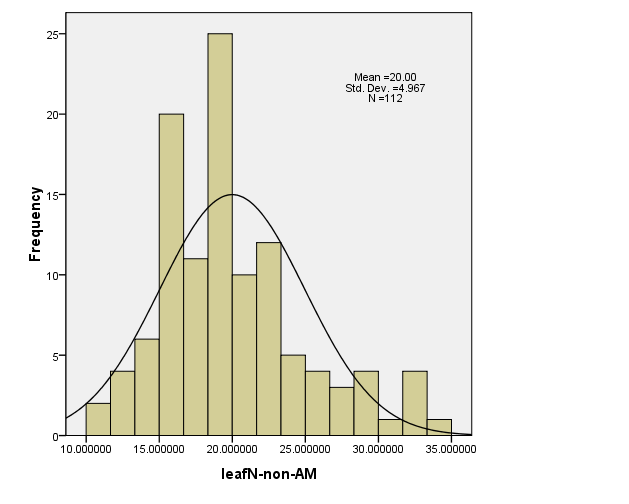


D

C


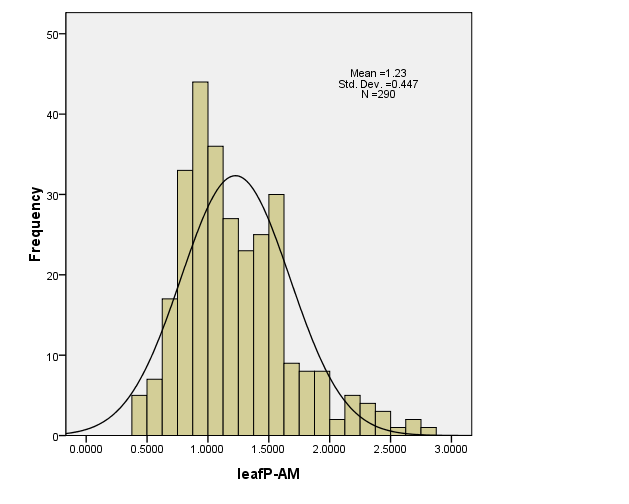

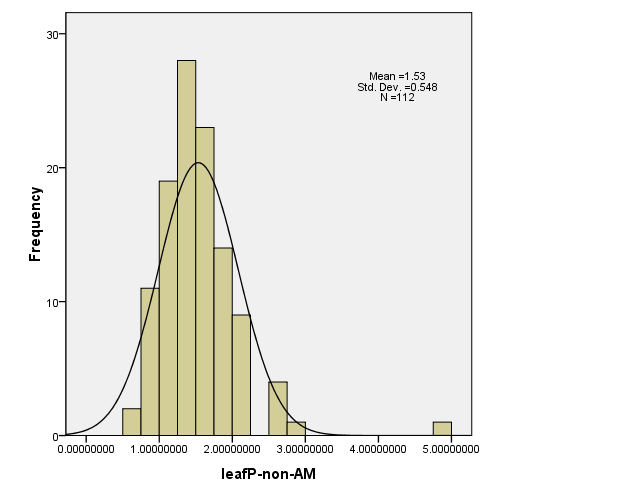


F

E

**Supplementary Figure S2** Density histogram of C, N, P concentration in leaves in AM and non-AM plants

AM plants: (A) leafC, (C) leafN, (E) leafP; non-AM plants: (B) leafC, (D) leafN, (F) leafP.


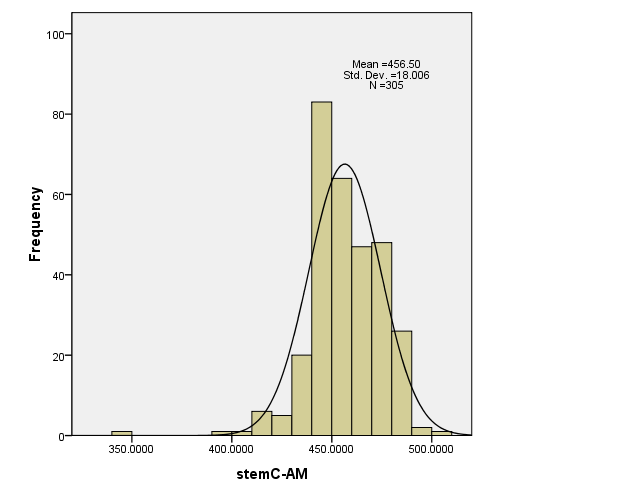

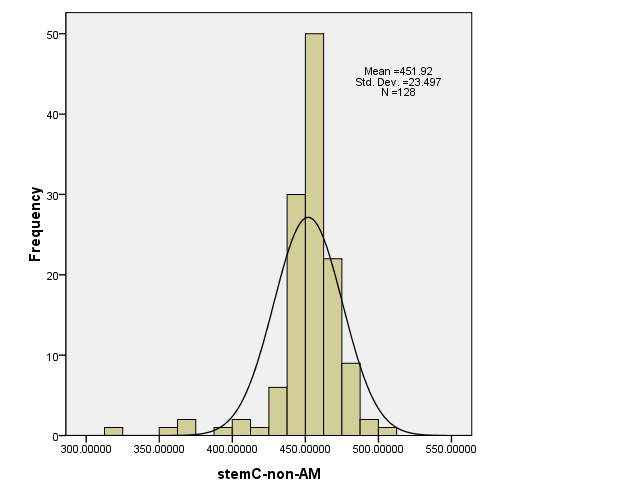


B

A


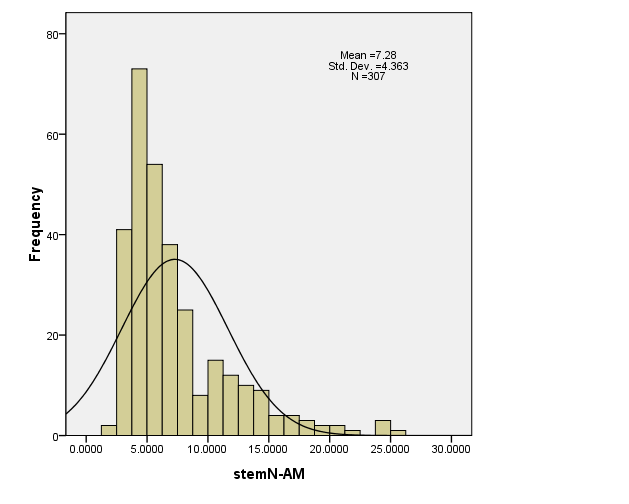

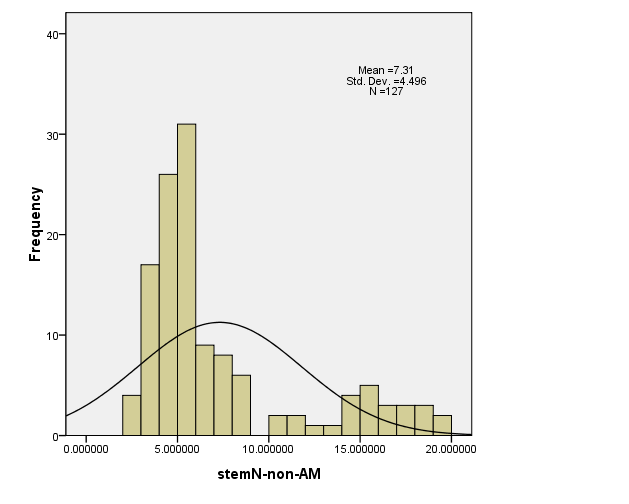


D

C


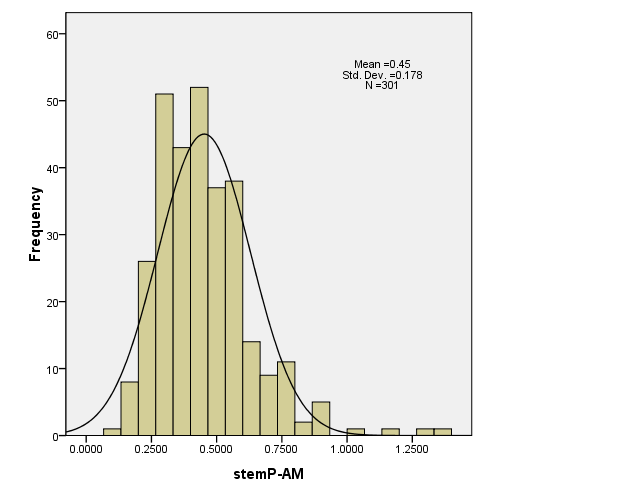

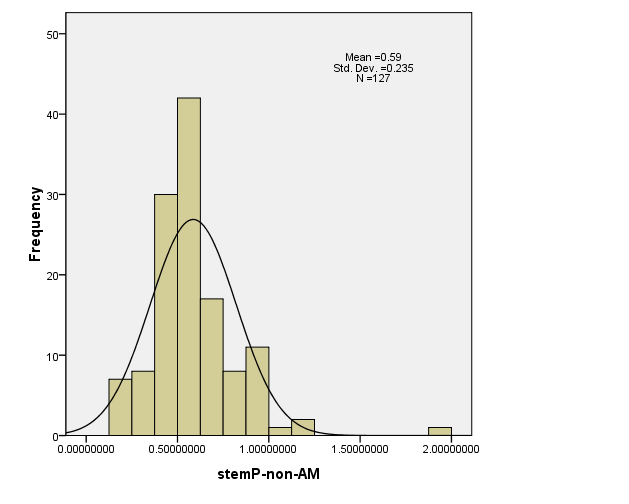


F

E

**Supplementary Figure S3** Density histogram of C, N, P concentration in stems in AM and non-AM plants

AM plants: (A) stemC, (C) stemN, (E) stemP; non-AM plants: (B) stemC, (D) stemN, (F) stemP.


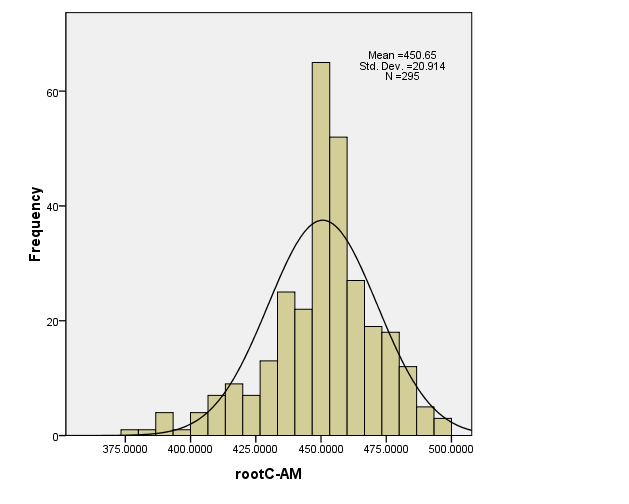

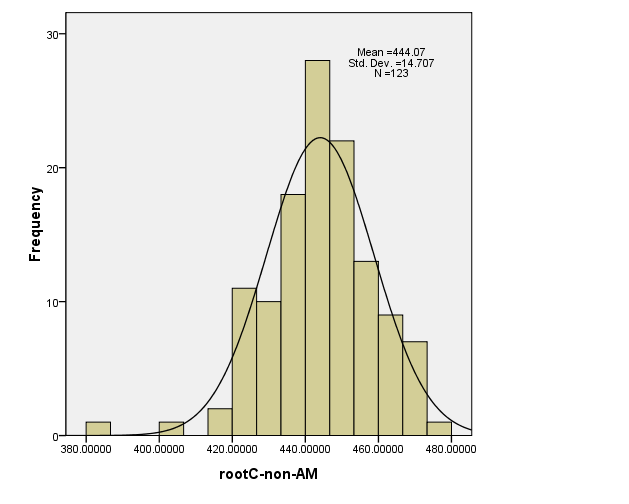


B

A


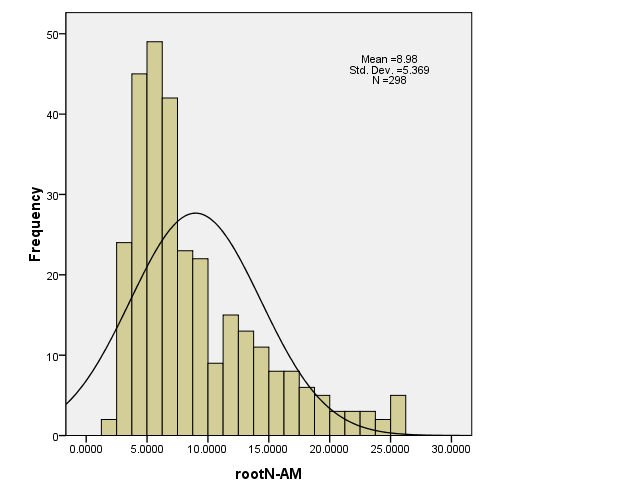

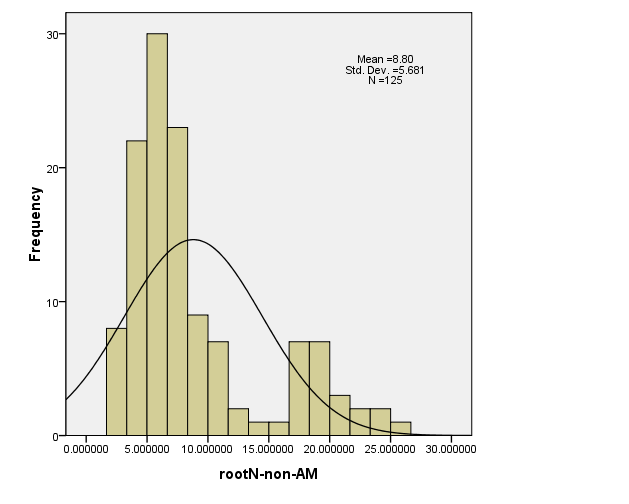


D

C


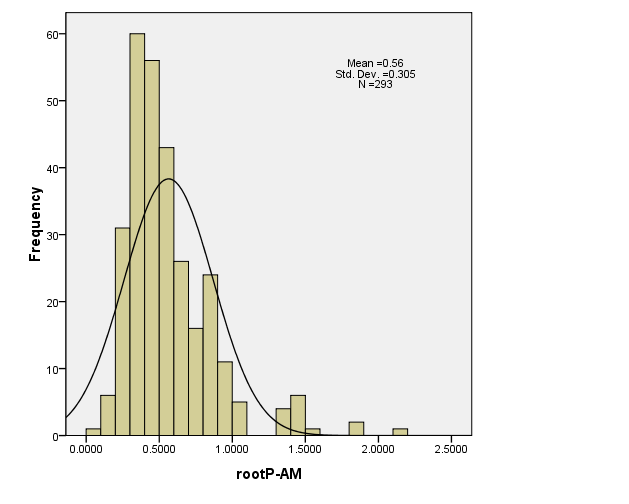

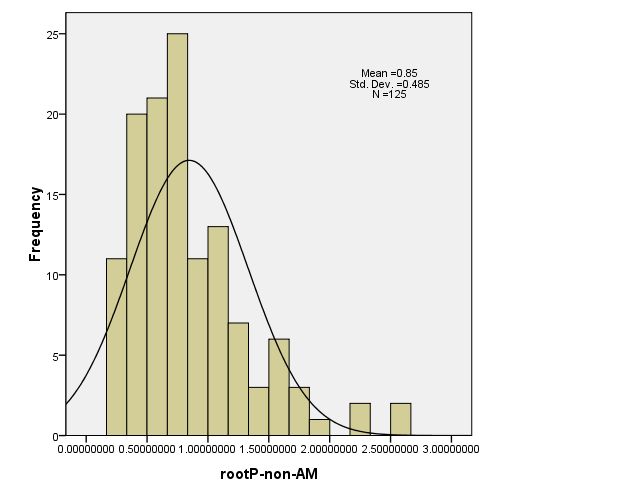


F

E

**Supplementary Figure S4** Density histogram of C, N, P concentration in roots in AM and non-AM plants

AM plants: (A) rootC, (C) rootN, (E) rootP; non-AM plants: (B) rootC, (D) rootN, (F) rootP.


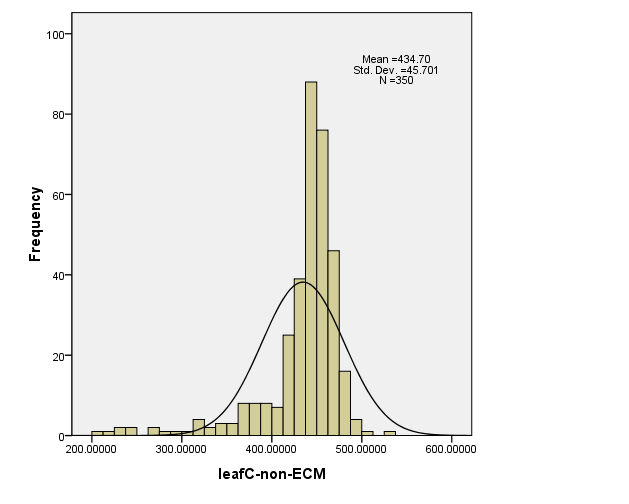

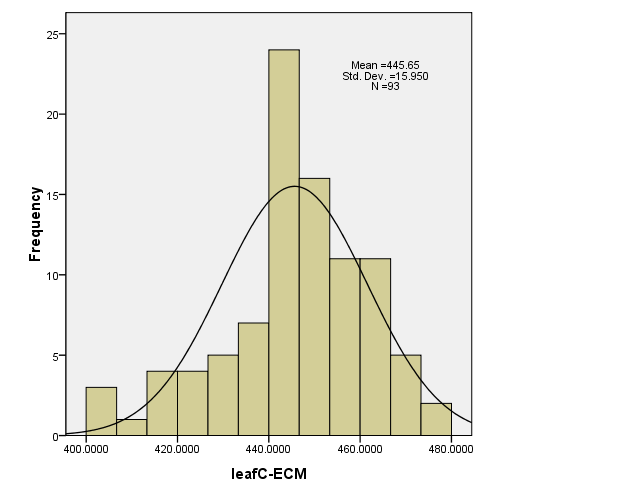


B

A


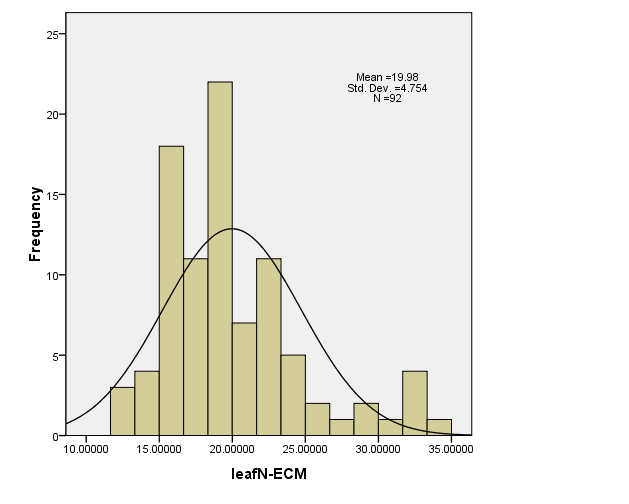

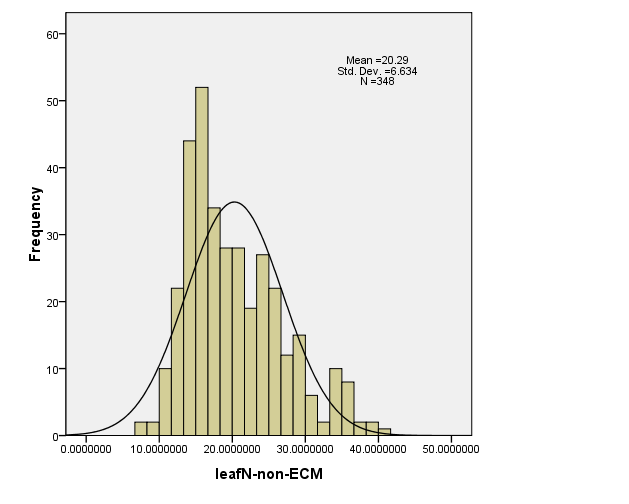


D

C


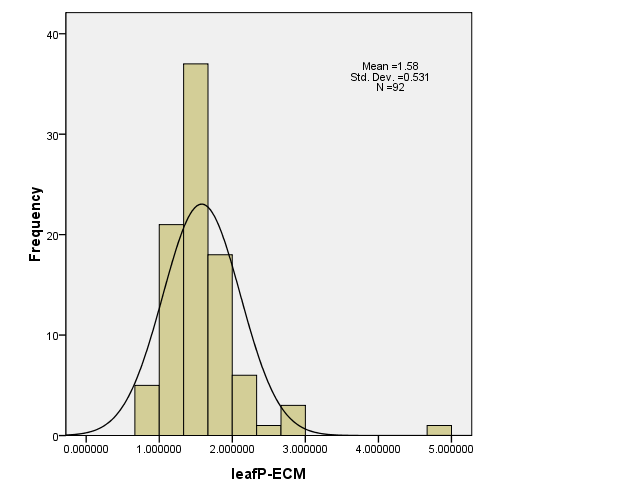

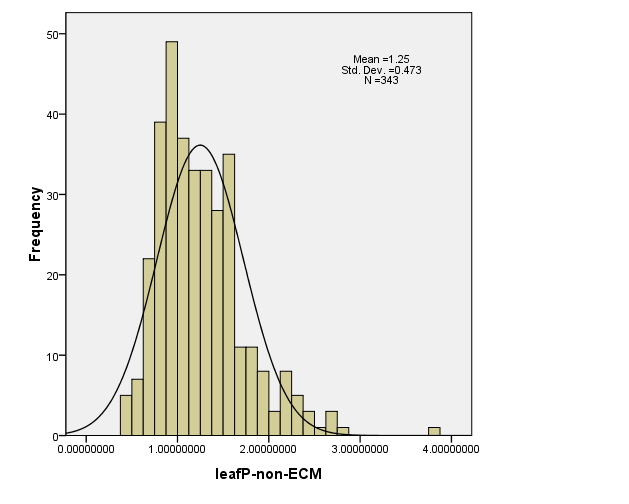


F

E

**Supplementary Figure S5** Density histogram of C, N, P concentration in leaves in ECM and non-ECM plants

ECM plants: (A) leafC, (C) leafN, (E) leafP; non-ECM plants: (B) leafC, (D) leafN, (F) leafP.


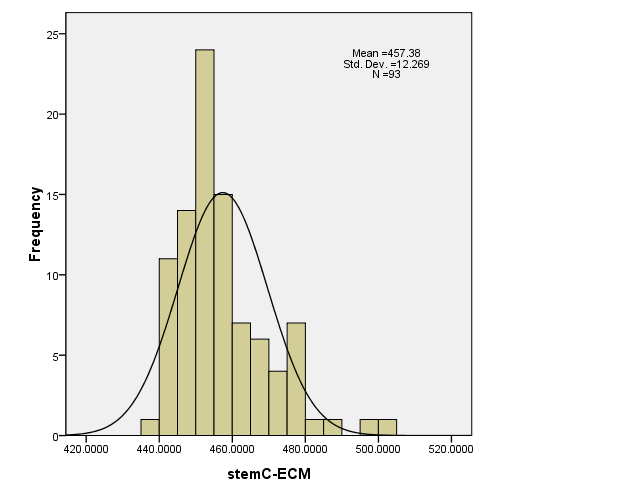

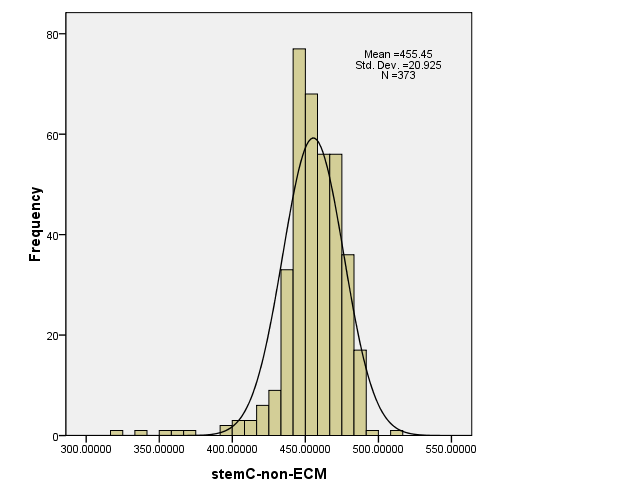


B

A


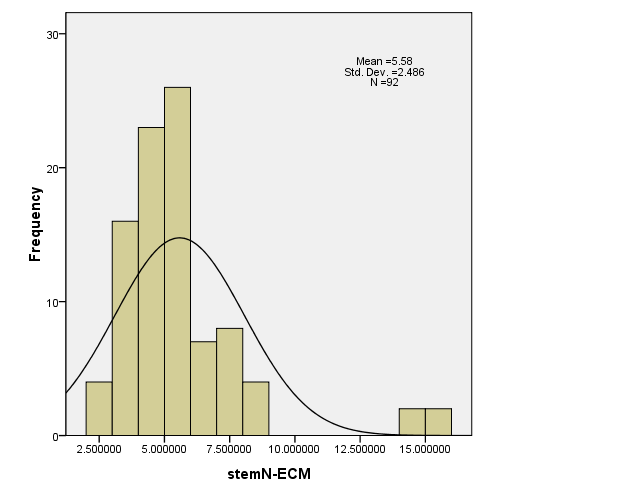

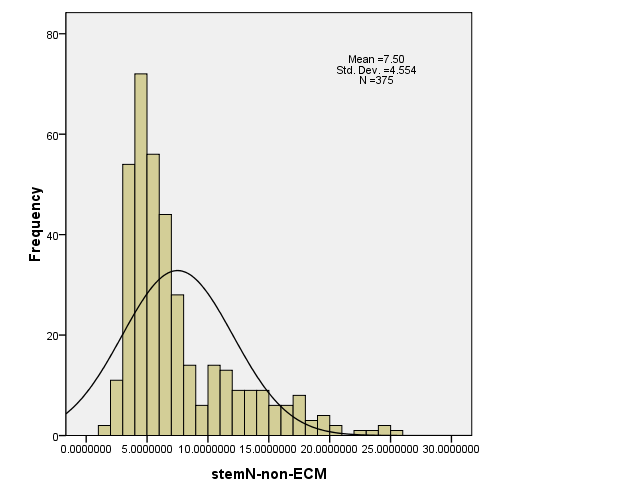


D

C


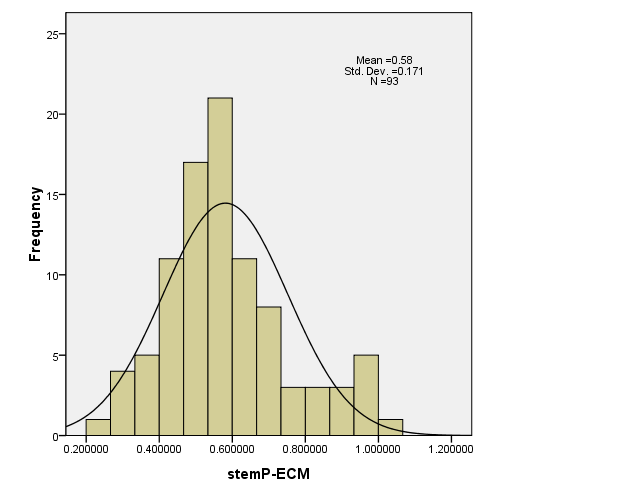

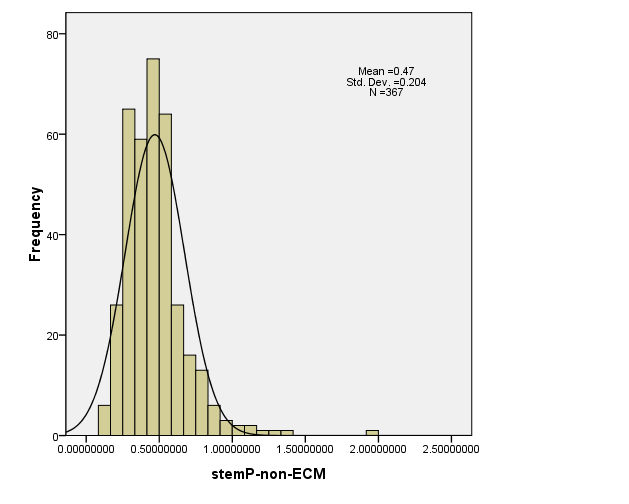


F

E

**Supplementary Figure S6** Density histogram of C, N, P concentration in stems in ECM and non-ECM plants

ECM plants: (A) stemC, (C) stemN, (E) stemP; non-ECM plants: (B) stemC, (D) stemN, (F) stemP.


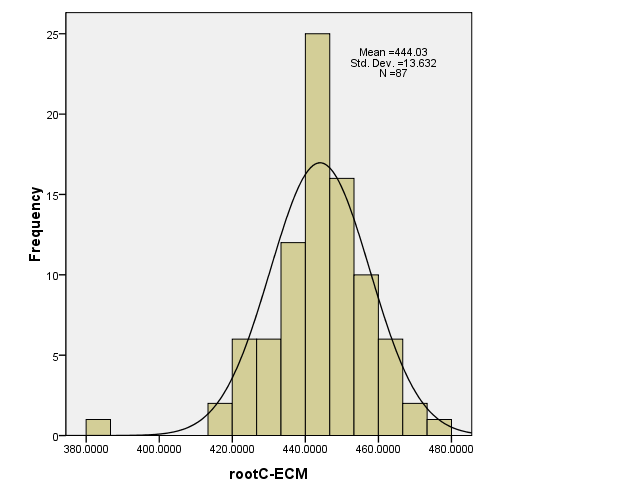

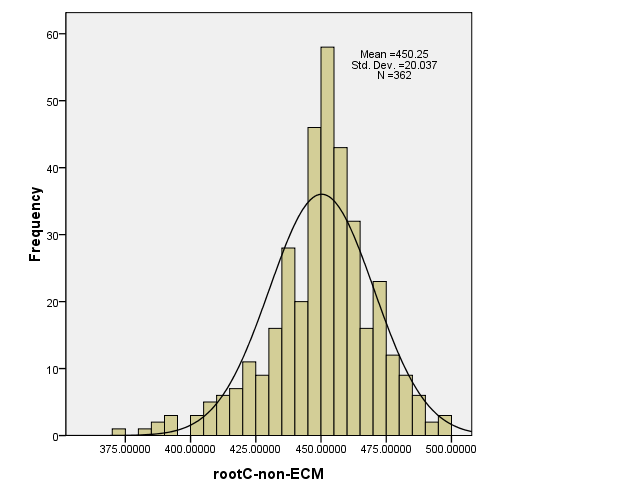


B

A


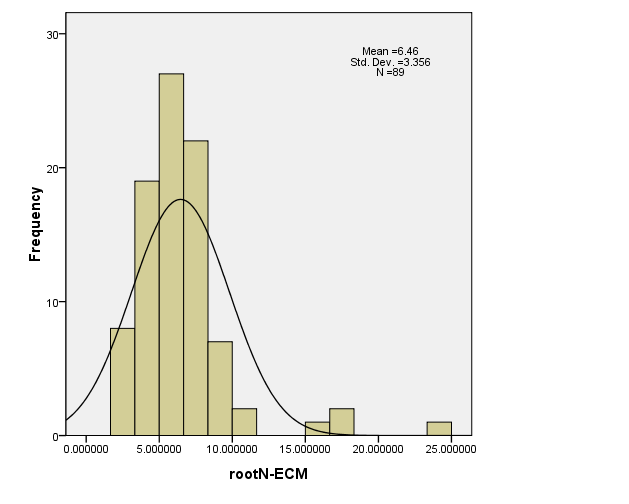

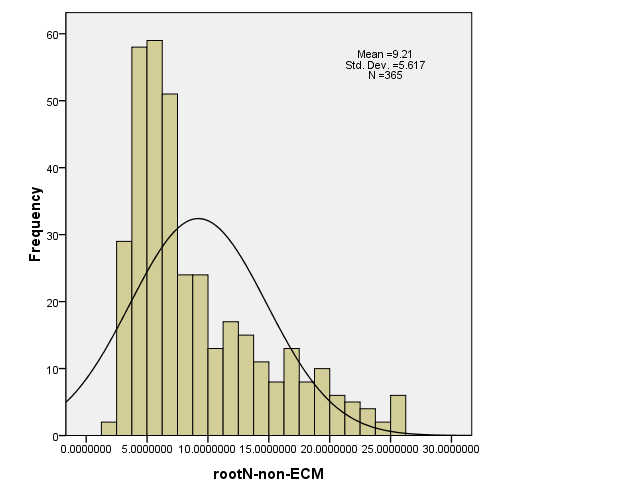


D

C


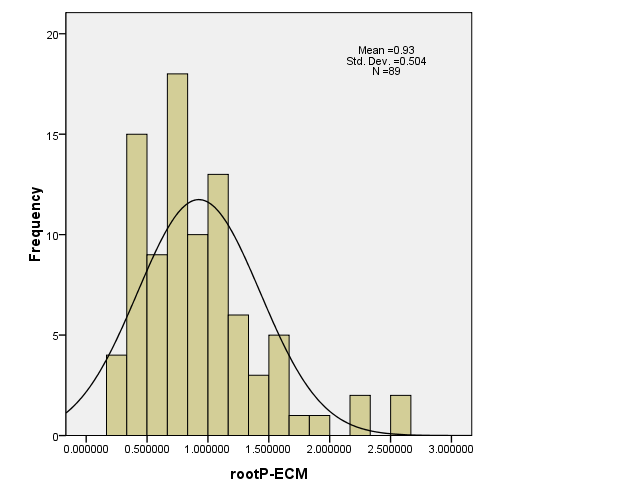

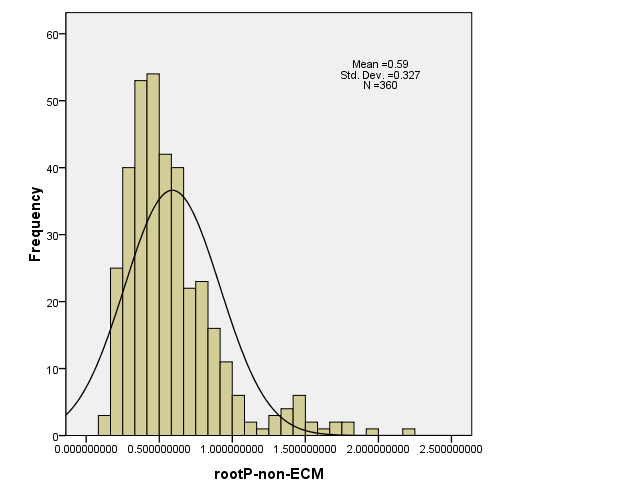


F

E

**Supplementary Figure S7** Density histogram of C, N, P concentration in roots in ECM and non-ECM plants

ECM plants: (A) rootC, (C) rootN, (E) rootP; non-ECM plants: (B) rootC, (D) rootN, (F) rootP.
